# Response by Lupon et al to “Artificial Intelligence in Plastic Surgery: A Bibliometric and Visual Analysis of the 100 Most-Cited English-Language Publications” by Mokhtar et al

**DOI:** 10.1093/asjof/ojag203

**Published:** 2026-10-15

**Authors:** Elise Lupon, Pharel Njessi, Olivier Camuzard

We read with interest the analysis by Mokhtar et al of the 100 most-cited English-language publications on artificial intelligence (AI) in plastic surgery, a field in which patient-facing large language model (LLM) evaluations are accumulating rapidly.^1,2^ Their dual independent screening, a priori taxonomy, and level-of-evidence grading provide a rigorous framework.

We previously reporteda contemporaneous Web of Science Core Collection analysis of the 50 most-cited articles on AI in plastic and reconstructive surgery, published in *Plastic and Reconstructive Surgery—Global Open*.^3^ Findings diverged markedly: Mokhtar et al identified patient education/LLM consultation (35%) and aesthetic surgery (34%) as dominant, whereas our cohort was led by imaging assessment (42%) and craniofacial surgery (64%) and contained no breast surgery articles (theirs: 21%).

Rank–citation curves illustrate the discordance (Figure 1). At Rank 1, their cohort is higher; from Ranks 2 to 50, ours is higher at each rank. Ninety of their 100 articles fall below our citation floor of 45, despite Scopus's broader coverage and a search 2 months later.

**Figure 1.**
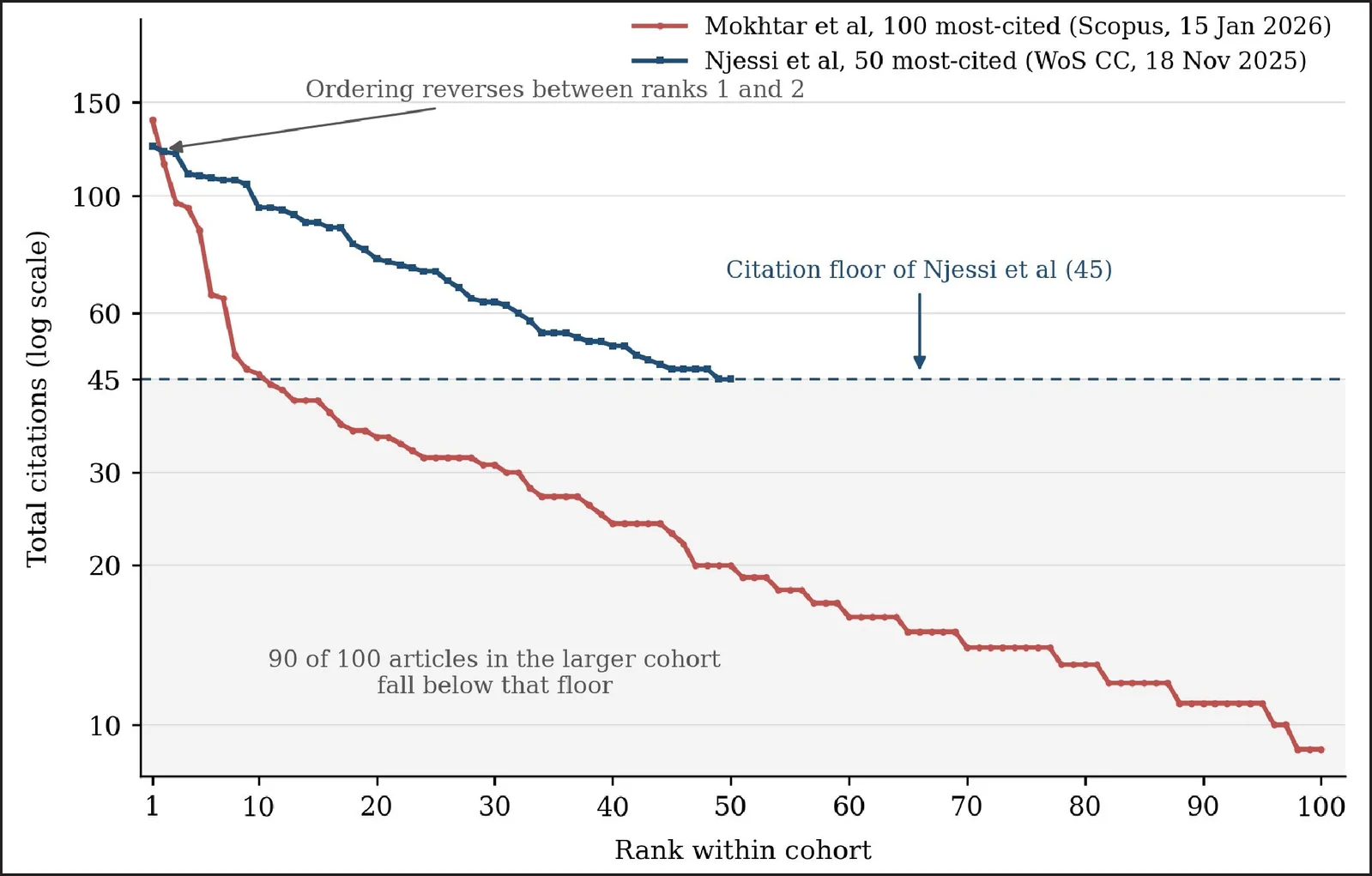
Rank–citation curves illustrate the discordance.

Differences in scope likely explain some omissions, notably craniomaxillofacial imaging papers outside plastic surgery journals but not the limited overlap. Cross-matching the supplementary lists identified only 5 of our 50 articles among their 100. Two apparently eligible LLM studies were absent: an evaluation of ChatGPT on the plastic surgery in-service examination exceeding 100 citations in our November 2025 search, and a rhinoplasty comparison of ChatGPT, BARD, and Bing AI published in this journal.^4,5^ Both fit their stated eligibility criteria and would have ranked within their top 10 using our earlier counts.

We suggest using citation floors as plausibility checks and sensitivity-testing candidate lists against a second database before drawing thematic conclusions. We thank the authors for their contribution.
